# Influence of Transfer Entropy in the Short-Term Prediction of Financial Time Series Using an ∊-Machine

**DOI:** 10.3390/e24081049

**Published:** 2022-07-30

**Authors:** José Crispín Zavala-Díaz, Joaquín Pérez-Ortega, Nelva Nely Almanza-Ortega, Rodolfo Pazos-Rangel, José María Rodríguez-Lelís

**Affiliations:** 1 Faculty of Accounting, Administration and Informatics, Universidad Autónoma del Estado de Morelos, Cuernavaca 62209, Mexico; 2 Tecnológico Nacional de México/CENIDET, Cuernavaca 62490, Mexico; jpo_cenidet@yahoo.com.mx (J.P.-O.); jose.rl@cenidet.tecnm.mx (J.M.R.-L.); 3 Tecnológico Nacional de México/IT de Tlalnepantla, Tlalnepantla de Baz 54070, Mexico; nnaortega@outlook.com; 4 Tecnológico Nacional de México/IT de Cd. Madero, Ciudad Madero 89440, Mexico; r_pazos_r@yahoo.com.mx

**Keywords:** financial series, Shannon entropy, transfer entropy, 62H10, 62M02, 62M10, 62P05

## Abstract

Predicting the values of a financial time series is mainly a function of its price history, which depends on several factors, internal and external. With this history, it is possible to build an ∊-machine for predicting the financial time series. This work proposes considering the influence of a financial series through the transfer of entropy when the values of the other financial series are known. A method is proposed that considers the transfer of entropy for breaking the ties that occur when calculating the prediction with the ∊-machine. This analysis is carried out using data from six financial series: two American, the S&P 500 and the Nasdaq; two Asian, the Hang Seng and the Nikkei 225; and two European, the CAC 40 and the DAX. This work shows that it is possible to influence the prediction of the closing value of a series if the value of the influencing series is known. This work showed that the series that transfer the most information through entropy transfer are the American S&P 500 and Nasdaq, followed by the European DAX and CAC 40, and finally the Asian Nikkei 225 and Hang Seng.

## 1. Introduction

Forecasting financial series, mainly the closing price of stocks, is a research area in different knowledge fields, from the financial field [1] to approximate solution methods such as neural networks [2] to forecasting using methods and algorithms based on Shannon entropy [3,4,5,6,7,8,9,10,11]. These models consider the historical data of the series to be calculated without considering explicitly the influence of other series or other stock markets for performing the forecast. To this end, it is assumed that the historical price already includes the influence of other series or financial markets.

The influence among financial markets is important because of the interaction of the behavior of the markets and the corresponding financial series. Therefore, it is important to have a knowledge of the financial series that influences another and whether the first one has a significant influence so as to modify the closing price of some stock. The analysis of the influence among financial markets is carried out through the interchange of information among financial series, where this is calculated using transfer entropy [12,13,14,15]. Transfer entropy may determine the relations among financial series, for example, the series that transmits the most information or which markets are the most dominant. With these analyses, financial crises have been detected and explained, from the originating market up to the effects on other financial markets.

Transfer entropy and its modifications are based on Shannon’s entropy calculation [16] by adding new terms of joint probability for considering the two financial time series [12]. The calculations of transfer entropy between the series are carried out by pairs, i.e., from a series **x** to a series **y** or vice versa [1,2], and the direction of the transfer entropy is important. Consequently, determining the series that best transfers entropy to another is a combinatorial problem because it implies calculating a number transfers given by the number of permutations Pr(*n*, 2), which is a factorial number of a complexity that is bounded by O(*n^n^*) [17], which makes the problem non-computable. For this reason, the analyses presented in [12,14] consider a reduced number of series; however, only in the work mentioned in [13] were 38 financial series considered. Therefore, a large amount of computational work is needed to approximate a work network and calculate the dynamics of the relation of the financial series [12,13,14,15].

Transfer entropy allows us to know what happened in previous periods and whether it was determined that there exists an interaction among the series and financial markets. Therefore, the following question arises: how can this interaction be used for improving or influencing explicitly the future behavior of a financial series? For answering this question, it is necessary to consider three issues. First, devise a method or modify one that considers transfer entropy. Second, determine the financial series that has the best influence on another series. Third, consider the transfer entropy taking into account the asynchrony of the opening and closing times of the financial markets due to the difference of time zones in the respective countries.

This research focuses on solving the first issue: devising a method that considers the transfer entropy and the historical values of the series for predicting future values. The prediction is for the short term, a 100-day period [1]. The initial model is an ∊-machine [11], which we used in a previous work. For showing the influence of one series on another, it is considered that the influencing series is known. The prediction is performed using the series of the closing values of six different markets: two from the American stock market, the S&P 500 and the Nasdaq; two from the European market, the CAC 40 and the DAX; and two from the Asian market, the Hang Seng and the Nikkei 225 [18]. In this first phase, all the permutations necessary for the six series are calculated.

**Figure 1:**
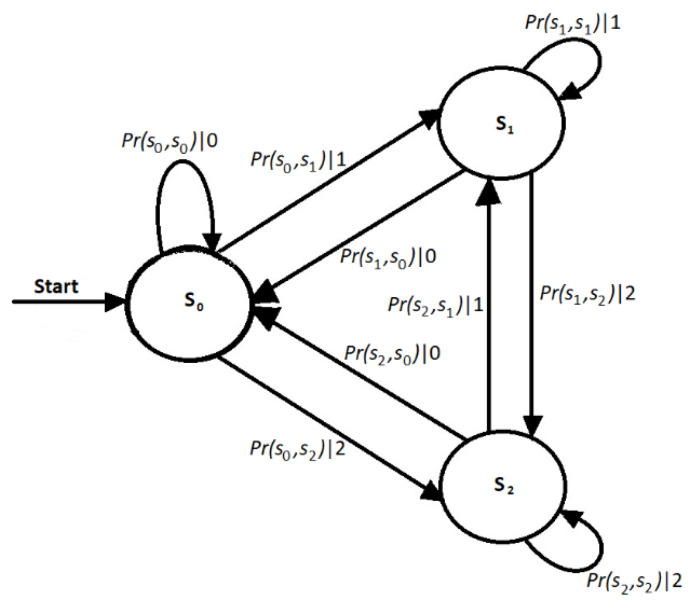
Example of the ∊-machine.

The ∊-machine [19] is represented in different ways according to the knowledge field where it is applied. In the computer science field, it is known as a stochastic finite state machine. For this objective, the first thing to do is to construct a probabilistic finite state machine (PFSM) with output [11], which is transformed into the ∊-machine when the conditional probabilities from one state to another are known, as shown in Figure 1.

For predicting closing prices with the ∊-machine, a procedure was designed for using the transfer entropy in case of ties for moving from one state to another. The transfer entropy is not added or subtracted from that calculated using the historical data for avoiding the introduction of an error because its origin is different.

For considering the influence of a series on another, it is necessary that the two financial series have the same length. Consequently, the same number of historical values is considered for all the series, which is 2000, although for some series, it is not the optimal value for obtaining the best estimation [8,11]. The historical values considered are from 1 July 2013 to 9 June 2021 and the prediction from 10 June 2021 to 29 October 2021 [18]. The predicted values with this method are measured with the real values of the series. For determining the series that has the best influence, an evaluation is performed with the following statistical metrics: mean absolute error (MAE), root-mean-squared Error (RMSE), mean absolute percentage error [MAPE], Theil’s inequality coefficient (Theil-U) and correct directional change (CDC) [1].

For the period of time analyzed, it was found that all the series can be influenced by two series, and the series that has the most transfer of information is the American S&P 500, with the second best being the Nasdaq, followed by the third best, where there was a tie between the European CAC 40 and the DAX, with the fifth best being the Asian Nikkei 225 and the last one the Hang Seng series. It important to make clear that this situation may be different if the analysis is carried out with other series or for another period of time. Determining the series that best influences another one can be posed as a combinatorial optimization problem, where a larger number of financial series can be considered.

Section 2 of this article describes the ∊-machine and the improvements applied to it for considering pairs of financial series, transfer entropy and the description of the process for estimating the integer number of the series. Section 3 shows the prediction of the closing values of the S&P 500 series and the determination of the most influencing series. The same section presents the results of the American, European and Asian financial series. The end of the section includes a discussion of the results, the conclusions of this work and future work. This article includes four appendices with complementary information. Appendix A includes the plots of the financial series considered. Appendix B presents the returns of each series. Appendix C shows the series of integers, and Appendix D contains the predictions for all the series.

## 2. Materials and Methods

The ∊-machine used is seen from the point of view of computer science, i.e., as a stochastic finite state machine that reads a sequence of characters, calculates the probability of their occurrence and predicts the chain of subsequent characters [19]. For predicting the subsequent states, the fundamentals established by Shannon are considered [19] as well as the criteria for selecting the following state. That is, selecting the state with the lowest entropy closest to the entropy of the previous state of the sequence. With these assumptions, a procedure was implemented based on the ∊-machine for predicting the closing price of stocks and was compared versus a financial prediction method [11]. The method based on the ∊-machine obtained better precision in all the cases.

In this section, a modification of our first model is proposed for considering two time series and calculating the transfer entropy from one series to the other. Measuring the precision of the results is performed directly with the real values, and the statistical measures previously defined are used. By using this approach, a comparison versus another method is not necessary.

Section 2 is divided into four parts. The first describes the construction of the ∊-machine from a probabilistic finite state machine (PFSM) with output [11], up to its diagram of transitions from one state to another, from where the values of the input and output functions are obtained. The second part describes the procedure for calculating the closing price of a stock using the ∊-machine, and specifically, some of the steps of this process have to be modified for introducing the changes. The third part describes the transfer entropy and the formulas that are used. The fourth and last part describes the new procedure for forecasting the time series, where the assumptions for obtaining the model are established.

An algorithm was implemented using the process described in this section. The algorithm was programmed in ANSI C and was executed on a computer with the following characteristics: 2 processors Intel Core 2 Duo E8200 at 2.66 Ghz, 4GB of RAM, a 500 GB hard disk, and a 64-bit Linux Fedora operating system.

### 2.1. ∊-Machine

The ∊-machine [19] can be obtained from a probabilistic finite state machine (PFSM) with output [11]. A finite probabilistic automaton with output is defined by the 7-tuple (*Σ*, *Q*, *M*, *P*(0), *F*, *f*, *g)*, where: *Σ* is the input alphabet; *Q* is the set of states, finite and not empty; *M* is the matrix of probability of transition between states; *P*(0) is the vector of the initial state, and it contains the probability of the initial state, with each state of *Q* associated with a probability of being the initial state; *F* ⊆ *Q* is the set of final states or acceptance (not empty); *f* is the input function that reads the elements of the alphabet; and *g* is the output function, which is a counter of the transition between the states. With this function, the probability matrix is constructed from the frequency of the element read by the function *f*.

Matrix *M* has the conditional probabilities of the series, which were constructed by reading the elements that constitute the series with the input function *f* and were counted with the output function *g*. For introducing the dynamics of the system, it is considered that the transition matrix has captured the relevant information for predicting the future, the history of vector **x** at time *t*. This can be represented by *x_:t_* = …, *x_t_*_−3_, *x_t_*_−2_, *x_t_*_−1_ and the future by the values *x_t_*_+1_, *x_t_*_+2_, *x_t_*_+3_, … by vector *x_:t’_*. These are related by the equivalence relation given by Equation (1) [19].

(1) $$x_{:t}\sim x_{:t^{\prime }}\overset{}{\Leftrightarrow }Pr\left(x_{i}|x_{:t}\right)=Pr\left(x_{i}|x_{:t^{\prime }}\right)$$

The equivalence relation ~ establishes that the causal states of Q, the space of each state and the transitions from state to state are the dynamics τ of the processes of the ϵ-machine. An outline of the ϵ-machine of the example is shown in Figure 1 [11].

Since the ∊-machine has discrete conditional probabilities, the entropy is directly calculated from transition matrix for obtaining the next state and, consequently, the number of the time series. The ∊-machine has important properties that can be found in reference [19].

### 2.2. Procedure for Determining Future Values with the ∊-Machine

The procedure used for predicting the series of values is described next [11]. To determine the series of values of the vector *x_:t′_*, it is required to select the next state *s_j_* from the *s_i_* state, given that a *s_i_* state can go to different *s_j_* states, as shown in Figure 1. Recent analyses of financial series have shown that they go through cycles, where entropy values can grow or decrease [15]. Changes in entropy are considered for the selection of the next state, *s_j_*, according to the following rules:

1. If the entropies of the destination states *j* are less than or equal to that of the source state *i*, the minor entropy closest to or equal to the entropy of the source *i* is considered, as established by Shannon [16].
2. If the entropies of the destination states *j* are greater than that of the source state *i*, the nearest major entropy is considered.
3. In case several different states have the same entropy, and in cases 1 or 2, the *s_j_* that is most likely to occur is selected. At this point is where the transfer entropy is introduced for selecting the next state, *s_j_*.
4. The count of each of the transitions is updated and its new conditional probability is calculated.

### 2.3. Transfer Entropy

For the transfer entropy [12], let X = {*x_tn_*, …, *x_t−k_*_+1_} be a stationary Markov process of order *k*, then this dominates the probability for observation X at time *t*+1; additionally, *x* is conditional of the *k* previous observations, such that *p*(*x_t_*_+1_|*x_tn_*, …, *x_t−k_*_+1_) = *p*(*x_t_*_+1_|*x_t_*, …, *x_t−k_*). If the previous values are known, the average number of bits necessary to encode the observation of the series is given by the following formula, where *log* represents *log*_2_:

(2) $$h_{x}\left(k\right)=-\underset{x}{\sum }p\left(x_{t+1},x_{t}^{\left(k\right)}\right)logp\left(x_{t+1}|x_{t}^{\left(k\right)}\right)$$

where $x_{t}^{\left(k\right)}=\left(x_{t},\ldots ,x_{t-k+1}\right)$.

The model can be scaled up for the bivariate case [12]:

(3) $$h_{XY}\left(k,l\right)=-\underset{x}{\sum }p\left(x_{t+1},x_{t}^{\left(k\right)},y_{t}^{\left(l\right)}\right)log\left(x_{t+1}|x_{t}^{\left(k\right)},y_{t}^{\left(l\right)}\right)$$

where *x_t_* and *y_t_* represent the discrete states at time *t* of X and Y, respectively. Additionally, $x_{t}^{\left(k\right)}$ and $y_{t}^{\left(l\right)}$ denote the bidimensional vectors of the two processes X and Y, respectively.

The transfer entropy is quantified by the flow of information from Y to X. The transfer entropy can be calculated by subtracting the information obtained from the last observation of X only to the last observation of the joint probability of X and Y, which is defined by [12]:

(4) $$TE_{Y\to X}\left(k,l\right)=h_{X}\left(k\right)-h_{XY}\left(k,l\right)$$

For facilitating the calculation of the transfer entropy, the following assignment is made: *k* = *l* = 1 [13]. Another representation of the transfer entropy is the so-called normal formula for the transfer entropy from Y to X, which is given by the following expression:

(5) $$NTE_{Y\to X}=\underset{i_{n+1},\;i_{n},j_{n}}{\sum }p\left(p_{n+1},i_{n},j_{n}\right)log\frac{p\left(i_{n+1},i_{n},j_{n}\right)p\left(i_{n}\right)}{p\left(i_{n+1},i_{n}\right)p\left(i_{n},j_{n}\right)}$$

where *i_n_* is the *n*-th element of the series of variable X and *j_n_* is the *n*-th element of the series of variable Y.

As can be observed, the two expressions can be used for calculating the transfer entropy from one series to another. However, since the data are obtained by reading the series, in this work the transfer entropy is calculated using Equation (4).

### 2.4. Procedure for Calculating the Influence of Entropy for Determining the New State in the ∊-Machine

The modifications in the calculation process are the following:

(a) Consider two ∊-machines, one for calculating series **x** and the other for series **y**. For series **y**, the same assumption used for series **x** is made, i.e.,
  (6) $$y_{:t}\sim y_{:t^{\prime }}\overset{}{\Leftrightarrow }Pr\left(y_{i}|y_{:t}\right)=Pr\left(y_{i}|y_{t^{\prime }}\right)$$
  where for the history, *y_:t_* = …, *y_t_*_−3_, *y_t_*_−2_, *y_t_*_−1_, and the future by the values *y_t_*_+1_, *y_t_*_+2_, *y_t_*_+3_, … through vector *y_:t′_*.
(b) It is assumed that the elements of the series are for the same day from day 1 to 2000.
(c) When reading the elements of the time series *x_:t_* and *y_:t_*, the joint probability matrix is constructed, **xy**.
(d) For measuring the influence of series **y** on series **x**, it is assumed that the vector of future values *y_:t’_* is known. Each value of the vector is used for the corresponding iteration, from the first to the 100th.
(e) Read and transform the financial series into a series of integer numbers. The elements of the series of integers are read until the last state *s_i_* of series *x_:t_* is reached as well as the last state of series *y_:t_*.
(f) Using the state *s_i_* of series **x**, the next state *s_j_* is determined considering the possible candidate states, which have a similar entropy among them. The candidate states are selected according to the original process.
(g) The transfer entropy is calculated for each candidate state according to the corresponding element of the iteration of vector *y_:t_*.
(h) From the candidate states that have the same entropy in series **x**, the one with the highest transfer entropy is selected. In case of a tie in entropy and in transfer entropy, the method proposed in the referred process is used.

Since the transfer entropy could be considered as noise in the signal, this is not added to the calculated entropy for going from a state, *s_i_*, to a state, *s_j_*. In the procedure described, both factors are considered separately.

## 3. Results

The closing prices of stocks and the returns that are calculated from the first ones are real numbers. Consequently, building a PFSM using the returns would be a non-computable problem due to the number of states that the state machine would need. For generating the characters that read and process the PFSM, a procedure described in the first part of this section is followed. For explaining the procedure, the series for the closing price of the S&P 500 is used.

The algorithm implemented was designed for executing a pair of closing price series at one time. The execution time of the algorithm is smaller than one second for each pair of series. The program was executed one time for each permutation of the series.

The comparison of results focuses on determining the influence of different stock market series on the closing price of a particular series. For each series, the influence of the other five series is determined. Additionally, the forecast of a series is calculated without considering the influence of any series. The results obtained are compared to the real values of the series. Therefore, it is not necessary to compare them using another method because statistical metrics give the precision of the results with respect to the real values and the objective is to know the influence of transfer entropy on the forecast of the financial series.

### 3.1. Prediction for the S&P 500 Series

The following sections show the elements for constructing the ∊-machine.

#### 3.1.1. Construction of the Elements of the PFSM

For constructing the ∊-machine, it is necessary to construct the probabilistic finite state machine (PFSM) with output [11], defined by the 7-tuple (*Σ*, *Q*, *M*, *P*(0), *F*, *f*, *g*). Therefore, the process starts with the definition of the input alphabet, *Σ*.

**Figure 2:**
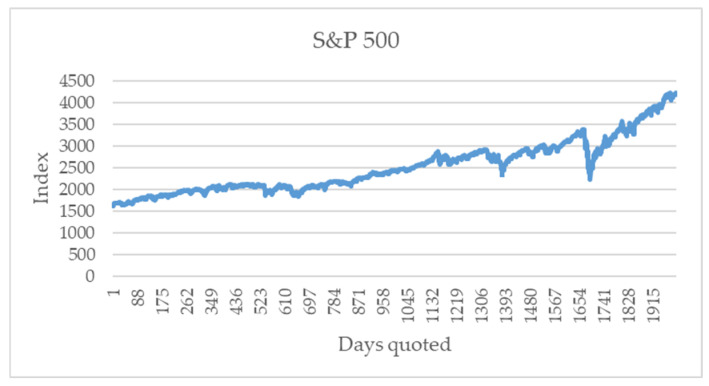
Closing value for S&P 500 from 1 July 2013 to 9 June 2021 (2000 data).

The alphabet is obtained from the return of the closing values variation. The series of the closing value for the S&P 500 is shown in Figure 2. These data are public [18].

In some research, the return of financial series is calculated using the difference of the price logarithms. In this work the return is calculated with the following formula [1,2]:

(7) $$R_{t}=\frac{\left(P_{t}-P_{t-1}\right)}{P_{t-1}}$$

where *P_t_* is the stock price at time *t*, and *P_t_*_−1_ is the price at time *t*−1.

**Figure 3:**
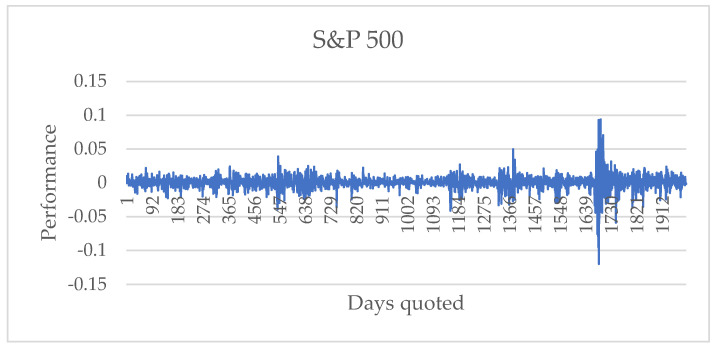
Return of the financial series S&P 500 from 1 July 2013 to 9 June 2021.

Using the prices of the series in Figure 2, the returns of the financial series S&P 500 are obtained, as shown in Figure 3.

Considering the returns of Figure 3 for the PFSM would make the problem non-computable because they are real numbers. Therefore, for constructing the alphabet of the PFSM from the returns of the financial series, the central limit theorem has to be considered [20]. If the returns of each of the series are grouped, they approximate a normal distribution. The area under the curve of the normal distribution is divided into two-percentile intervals, and each interval is represented by an integer. The set selected consists of 50 intervals [11], which defines the alphabet, *Σ* = {1, 2, 3, …, 50}.

**Figure 4:**
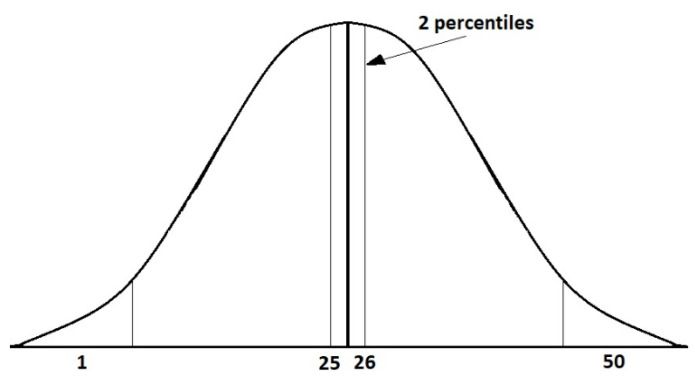
Plot of the normal distribution divided into two-percentile intervals.

Unlike the procedure of reference [11], where odd numbers were assigned to negative returns and even numbers to positive ones, here a change is made: negative returns are assigned a number from 1 to 25, while positive ones are assigned a number from 26 to 50, as shown in Figure 4.

This change is made because, in the previous assignment, if a number changed from 4 to 5, it indicated that a positive return changes to a negative one, instead of indicating that the change was negligible. For this reason, the way of numbering the percentiles was changed.

**Figure 5:**
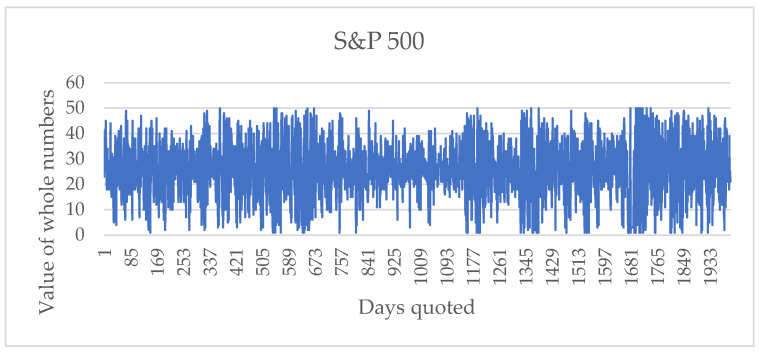
Sequence of integers representative of the returns of the S&P 500 series from 1 July 2013 to 9 June 2021.

When applying the process described, the sequence of integer numbers for the series S&P 500 is obtained, which is shown in Figure 5.

The sequence of integer numbers follows the sequence of the returns, where the axis of the location of zero, in this case, lies between numbers 25 and 26.

The remaining terms of the 7-tuple of the PFSM are defined. When the integers are defined, the number of states *Q* is also defined because, in the sequence of numbers, from any state another available state can be reached. Therefore, the set of states is defined *Q* = {*s*_1_, *s*_2_, *… s*_50_}. This number of elements generates a 50 × 50 probability matrix, *M*, which gathers the main characteristics of the 2000 history elements used. Function *f* reads the path defined by the 50 states of Figure 5. The output function *g* calculates the probabilities *Pr*(*s*_1_, *s*_1_), *Pr*(*s*_1_, *s*_2_), …, *Pr*(*s*_50_, *s*_50_). All the states have the same probability of being the initial state because the series can start from any state *P*(0) = {*s*_1_(1/50), *s*_2_(1/50), …, *s*_50_(1/50)}. All the states can be final states because any element of the alphabet can be the final state, *F* = {*s*_1_, *s*_2_, …, *s*_50_}. With this definition, the PFSM is constructed, which is then transformed into the ∊-machine [11].

#### 3.1.2. Data and Predictions of the S&P 500 Series

The data available for the series S&P 500, Nasdaq, CAC 40, DAX, Han Seng and Nikkei 225 are 2000 data points and are from 1 July 2013 to 9 June 2021 and are public [18]. The prediction is for 100 days, considered short-term [1], and it is from 10 June 2021 to 29 October 2021. By applying the process described in Section 2.4 for the S&P 500 series, the results described next are obtained.

**Figure 6:**
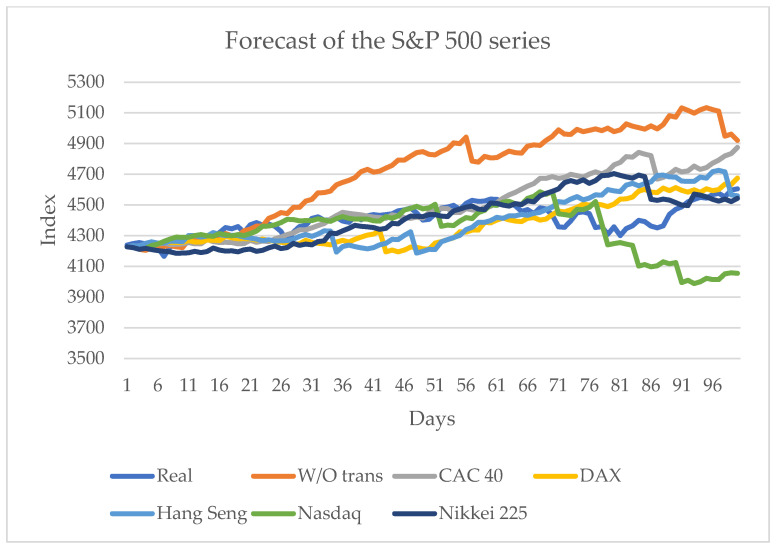
Forecast for the S&P 500 series considering the transfer entropy from 10 June 2021 to 29 October 2021.

Figure 6 shows that, if the index of any of the aforementioned series is known, the prediction is improved if it is compared to the prediction performed without considering the transfer entropy. Each of the series transfers information to the series in question to influence the selection of the subsequent states in the ∊-machine. For determining the series that best influences series **x**, the metrics of Table 1 are used [1].

To make the comparisons of the results obtained, the following statistical metrics are used: MAE, RMSE, MAPE, Theil-U and CDC. The statistics MAE and RMSE are measures dependent on the scale and allow a comparison between the real and predicted values; since these are closer to zero, the accuracy of the forecast will be better. When it is more important to evaluate forecast errors independently of the scale of the variables, MAPE and Theil-U are used, since they are constructed to have a value within the range of [0, 1], and zero indicates a perfect fit. CDC indicates sign change, and when the forecast equals the real value, CDC equals 1; otherwise, it equals 0. Their equations are shown in Table 1 [1].

These metrics are applied to the predictions considering the influence of other financial series.

Table 2 shows that the MAE and RMSE indicators are close to zero, and their lowest values occur when the transfer of information of the CAC 40 series is considered. Concerning the MAPE and Theil-U indicators, for Theil-U the best value was obtained for the same CAC 40 series, and for MAPE the best value was attained when no transfer entropy was considered (the first row). The other European series, the DAX, influences the S&P 500 series for accurately forecasting the sign change a bit above 50 (the third row).

**Figure 7:**
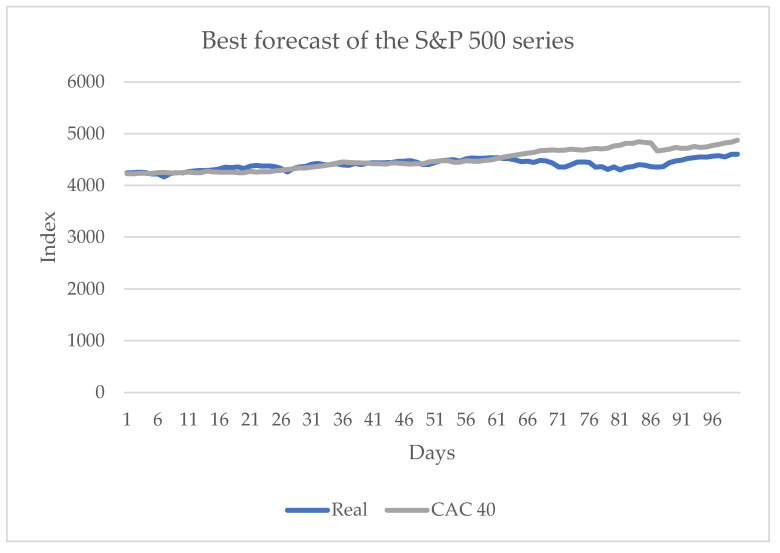
Forecast for the S&P 500 series when the values of the CAC 40 series are known, from 10 June 2021 to 29 October 2021.

Table 2 shows in bold face the best (smallest) values obtained. It shows that the best results are obtained when the closing values of the CAC 40 series are known; of the five metrics, three yield the best approximations. Another of the best approximations occurs when a forecast does not consider the transfer entropy, and the other is the sign change, which is obtained when the value of the DAX series is known. The best approximation is when the European series are known. The influence of the other American series, the Nasdaq, is not as significant as that of the European series; however, the results did improve when compared to the one without transfer entropy. Considering the preceding results, Figure 7 shows the best approximation.

Like the S&P 500 series, the calculations were performed for the financial series. Appendix A includes the plots of the financial series considered. Appendix B presents the returns of each series. Appendix C shows the series of integers, and Appendix D contains the predictions for all the series.

Next, the best predictions for the financial series are shown.

### 3.2. Prediction for the Nasdaq Series

The data for this series are in the corresponding Appendices. Table 3 shows the forecasts using the other series.

**Figure 8:**
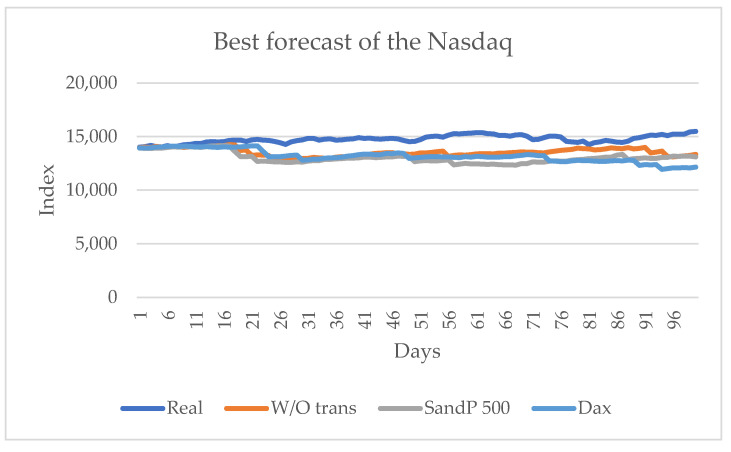
Forecast for the Nasdaq series when the values of the S&P 500 and DAX series are known.

Considering the real values of the series, the series that influences most significantly is the S&P 500 with two metrics and without considering any series with two metrics and, finally, the DAX series with one metric. Figure 8 shows these results graphically. In this series, knowing the closing price of the other American series, the S&P 500 series, and the European DAX series does have a significant influence.

### 3.3. Prediction for the DAX Series

The data for this series can be found in the corresponding appendices. Table 4 shows the forecasts using the other series.

**Figure 9:**
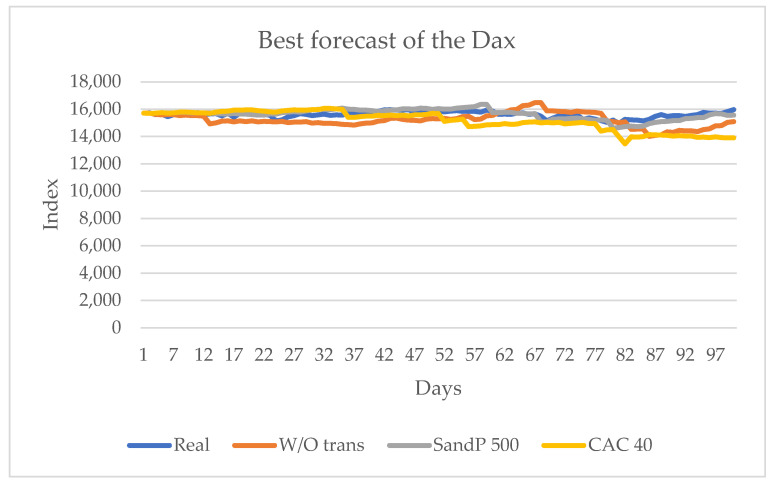
Forecast for the DAX series with the values of S&P 500 and CAC 40, and without the influence of another series from 10 June 2021 to 29 October 2021.

Table 4 shows that two are the best approximations to the real value. The first does not consider the influence of any series, and the second considers the influence of the S&P 500 series. It is important to mention that, when the influence of the other European series is considered, the best prediction of sign change is obtained. Figure 9 shows these results graphically.

As shown in Figure 9, the CAC 40 series influences the sign change of the DAX series, both European series. However, the one that may have the most information flow is the American S&P 500.

### 3.4. Prediction for the CAC 40 Series

The data for the tests are in the corresponding appendices, and Table 5 shows the values that were obtained for the statistical metrics.

**Figure 10:**
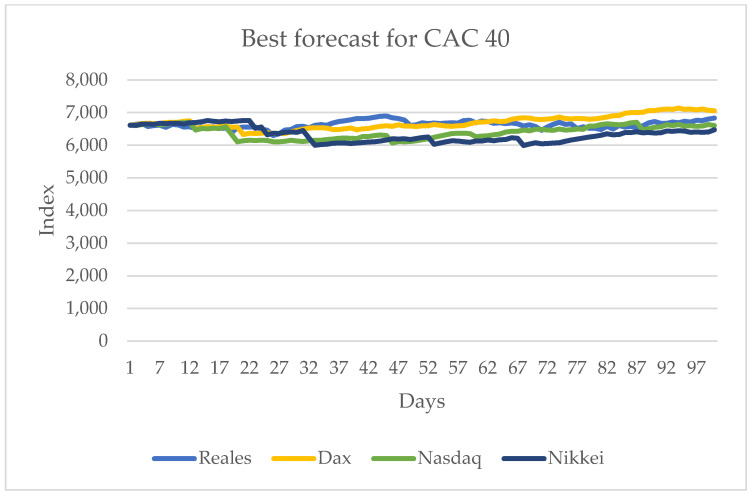
Forecast for the CAC 40 series with the values of DAX and Nikkei 225 from 10 June 2021 to 29 October 2021.

The results show that the DAX and Nikkei 225 are the ones that have the largest influence on the CAC 40 series. It is important to mention that the DAX is in the European market and the Nikkei 225 is in the Asian one. The other series, the Nasdaq, also has influence, but, unlike the ones previously mentioned, it is not significant. Figure 10 shows the series with the best results.

### 3.5. Prediction for the Hang Seng Series

The corresponding appendices present the data used for the closing values of the Hang Seng index, and Table 6 shows the statistical metrics for measuring the influence of the other series considered in this work.

**Figure 11:**
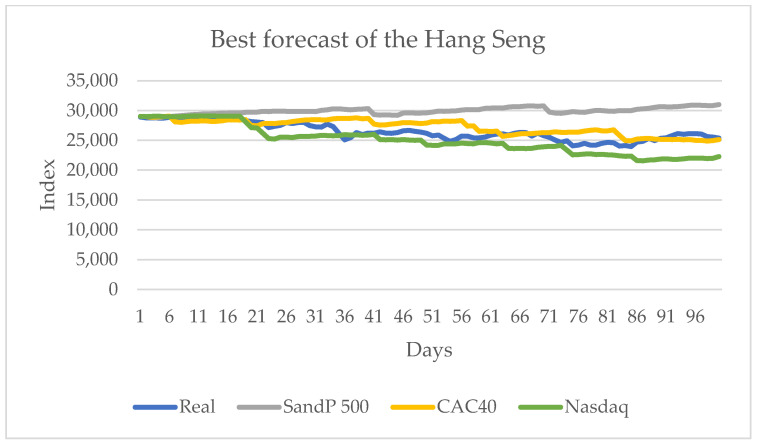
Forecast for the Hang Seng series with the values of S&S 500, Nasdaq and CAC 40 from 10 June 2021 to 29 October 2021.

Table 6 shows that the series that transmit more information are the American series, the S&P 500 and the Nasdaq, with the first one with three indicators and the second with two. A European series that influences the sign change (indicated by CDC) is the European series CAC 40. In this series, for all cases, the influence of knowing the value of any series improves the forecast obtained without considering transfer entropy. Figure 11 shows the series with the best results.

The other Asian series, the Nikkei 225, does not show a significant influence on the selection of the next state in the Hang Seng series. The American series, the S&P 500 and the Nasdaq, are the ones that have the most transfer of information for selecting the next state in the forecast.

### 3.6. Prediction for the Nikkei 225 Series

The corresponding appendices contain the data used for the Nikkei 225 closing values, and Table 7 shows the statistical metrics for measuring the influence of the other series considered in this work.

**Figure 12:**
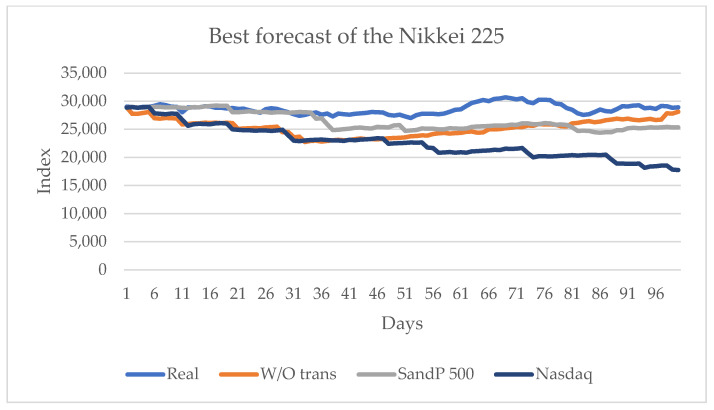
Forecast for the Nikkei 225 series with the values of S&P 500 and Nasdaq and without transfer entropy, from 10 June 2021 to 29 October 2021.

Table 7 shows that the series that transmits more information is the American series S&P 500, and with the influence of the other American series, the Nasdaq, the highest sign change is obtained (indicated by CDC). For this series, one of the indicators obtains the best value when no influence of any other series is considered. Figure 12 shows the series with the best results.

For this series, the transfer of information from the Americas series, the S&P 500 and the Nasdaq, is higher than that from the European series; the Asian series have no significant influence.

## 4. Discussion

The results show that it is possible to modify the procedure for predicting future values of the closing value of stocks using the ∊-machine by considering the transfer of information calculated as the transfer entropy of another financial series to the closing value.

The knowledge of a financial series beforehand influences the stock prices. All the series considered have influences of different magnitudes. In some cases, the influence is more significant for the selection of the subsequent state of the ∊-machine.

The results show that the American financial market is the one that transmits more information, which coincides with other researchers [15] who have found that the American market is the one that transmits the most information, and the Asian market is the one that transmits the least information. Table 8 shows the number of times that the transfer entropy of certain series obtained the best approximation for another series when using the ∊-machine.

The results in Table 8 show that the American series, the S&P 500, is the one that transmits the most information among the series. The second best are the American Nasdaq and the European DAX. The European series CAC 40 occupies the third place, the Asian series Nikkei 225 is in fourth place, and the last place is for the Asian series Hang Seng.

In Table 8, it is observed that the European series CAC 40 transmits the most information to the American series S&P 500. In turn, this European series is influenced by the Asian Nikkei 225 and the American Nasdaq, which shows that, in some sense, series from different markets are interconnected. This means that the necessary analysis includes not only the first series that best transmits information since it is possible to find in a subsequent stage some result that influences the first observation.

The results obtained are a simulation of a future forecast, and considering the price history of the series considered, similar results to those obtained by other researchers were obtained [15]: the dynamics of the financial series determined in the recent past is the same as in the present and perhaps in the future.

## 5. Conclusions

It is concluded that the objective proposed for this research was successfully achieved because of the following reasons: (1) a procedure was designed that is based on the ∊-machine, the rules established by Shannon and the rules proposed in this work for calculating transfer entropy; and (2) the calculation of entropy uses the historical values of the series to be calculated.

From the assumptions considered (first, knowing the future values of the series of values *y_t’_*, and second, the calculation of the transfer entropy independently from the calculation process of the ∊-machine), it is concluded that it is possible to influence the selection of the next state *s_j_* and, consequently, the stock market index **x**.

The calculation of the transfer entropy using the ∊-machine implies the calculation of all the permutations for determining which one or which ones of the financial series influence the obtaining of a better forecast. Additionally, if it is necessary to introduce in the calculation a larger number of financial series, it is necessary to formulate the problem as a combinatorial optimization problem whenever the dynamics of the financial series is a combinatorial optimization problem.

For obtaining the results of this work, it was assumed that vector *y_t’_* of the influencing series was known, and the next step was to determine the series prices without knowing this vector, but, following the closing hours of the financial markets, to calculate the influence on the stock markets that are still open. To formulate this problem, it is necessary to know which is the initial market and then the series that will be calculated.

## Figures and Tables

**Table 1:** Statistical performance measurement metrics.

Metric	Description	
Mean absolute error	$MAE=\frac{1}{T}\sum_{t=1}^{T}\left|\tilde{y_{t}}-y_{t}\right|$	(8)
Mean absolute percentage error	$MAPE=\frac{100}{T}\sum_{t=1}^{T}\left|\frac{\tilde{y_{t}}-y_{t}}{y_{t}}\right|$	(9)
Root-mean-squared error	$RMSE=\sqrt{\frac{1}{T}\sum_{t=1}^{T}{\left(\tilde{y_{t}}-y_{t}\right)}^{2}}$	(10)
Theil’s inequality coefficient	$\mathrm{Theil}-U=\frac{\sqrt{\frac{1}{T}\sum_{t=1}^{T}{\left(\tilde{y_{t}}-y_{t}\right)}^{2}}}{\sqrt{\frac{1}{T}\sum_{t=1}^{T}{\left(\tilde{y_{t}}\right)}^{2}}+\sqrt{\frac{1}{T}\sum_{t=1}^{T}{\left(y_{t}\right)}^{2}}}$	(11)
Correct directional change	$CDC=\frac{100}{N}\sum_{t=1}^{N}D_{t}$ $\mathrm{Where}\;D_{t}=1$ $,\;\mathrm{if}\;\tilde{y_{t}}y_{t}>0$ $,\;\mathrm{else}\;D_{t}=0$	(12)
$\mathrm{where}:\;y_{t}$ is the real change at time *t*. $\tilde{y_{t}}$ is the forecast of change at time *t*. *t* = 1 to *t* = *T* for the forecast period.		

**Table 2:** Metrics for the prediction of the S&P 500 series.

	MAE	MAPE	RMSE	Theil-U	CDC
W/O trans	0.007046	**331.679688**	0.009233	0.680105	48.484848
CAC40	**0.006208**	378.035461	**0.007989**	**0.674232**	46.464645
DAX	0.006298	488.436951	0.008612	0.729542	**50.505051**
Hang Seng	0.006900	529.977722	0.009972	0.728371	46.464645
Nasdaq	0.007297	373.871674	0.010945	0.703083	48.484848
Nikkei	0.006494	449.047852	0.008233	0.686128	49.494949

**Table 3:** Metrics for the prediction of the Nasdaq series.

	MAE	MAPE	RMSE	Theil-U	CDC
W/O series	0.008851	**458.042206**	0.013113	0.736870	**53.535355**
S&P 500	**0.008377**	817.115417	**0.012844**	0.726994	48.484848
CAC 40	0.009395	585.322693	0.013516	0.721487	51.515152
DAX	0.008759	871.118896	0.012935	**0.714895**	52.525253
Hang Seng	0.011798	562.663330	0.016994	0.746129	50.505051
Nikkei	0.010775	776.217163	0.016060	0.791978	40.404041

**Table 4:** Metrics for the prediction of the DAX series.

	MAE	MAPE	RMSE	Theil-U	CDC
W/O series	0.009036	**618.376221**	0.011943	**0.650501**	52.525253
S&P 500	**0.007796**	844.586304	**0.010715**	0.676640	50.505051
CAC 40	0.008825	892.767029	0.013338	0.717789	**57.575756**
Hang Seng	0.013858	662.613037	0.018884	0.695072	56.565655
Nasdaq	0.009343	751.972229	0.014088	0.726485	51.515152
Nikkei	0.009064	1206.864868	0.013062	0.668989	48.484848

**Table 5:** Metrics for the prediction of the CAC 40 series.

	MAE	MAPE	RMSE	Theil-U	CDC
W/O series	0.008694	6552.129395	0.011883	0.699467	54.545456
S&P 500	0.009227	9994.777344	0.012657	0.734951	43.434345
DAX	**0.008055**	6439.956055	**0.010542**	0.689794	47.474747
Hang Seng	0.009337	5906.416992	0.012740	0.690702	48.484848
Nasdaq	0.008656	6040.721191	0.012272	**0.680308**	46.464645
Nikkei	0.009105	**2575.624023**	0.012615	0.681498	**59.595959**

**Table 6:** Metrics for the prediction of the Hang Seng series.

	MAE	MAPE	RMSE	Theil-U	CDC
W/O series	0.014074	561.181519	0.018278	0.766479	40.404041
S&P 500	**0.010924**	1146.378662	**0.014252**	0.737163	**54.545456**
CAC 40	0.012453	1585.553955	0.016222	0.680928	**54.545456**
DAX	0.016491	963.591003	0.020860	0.716018	43.434345
Nasdaq	0.011971	**525.337402**	0.016503	**0.664015**	51.515152
Nikkei	0.014792	663.99981	0.019339	0.686714	49.494949

**Table 7:** Metrics for the prediction of the Nikkei 225 series.

	MAE	MAPE	RMSE	Theil-U	CDC
W/O series	0.011474	**377.160492**	0.015506	0.656048	55.555557
S&P 500	**0.009853**	1154.495117	**0.013700**	**0.640071**	50.505051
CAC 40	0.010712	996.726318	0.015071	0.680227	52.525253
DAX	0.013742	603.969604	0.019325	0.708990	47.474747
Hang Seng	0.015252	1165.947144	0.021328	0.744763	53.535355
Nasdaq	0.011941	546.979126	0.017283	0.643839	**56.565655**

**Table 8:** Number of times of largest transmission of information from one series to another.

	Influencing Series					
Influenced Series	S&P 500	Nasdaq	CAC 40	DAX	Hang Seng	Nikkei 225
S&P 500	1	0	**3**	1	0	0
Nasdaq	2	2	0	1	0	0
CAC 40	0	1	0	2	0	2
DAX	2	0	1	2	0	0
Hang Seng	**3**	2	1	0	0	0
Nikkei 225	**3**	1	0	0	0	1
**Total**	**11**	**6**	**5**	**6**	**0**	**3**

**Figure A1:**
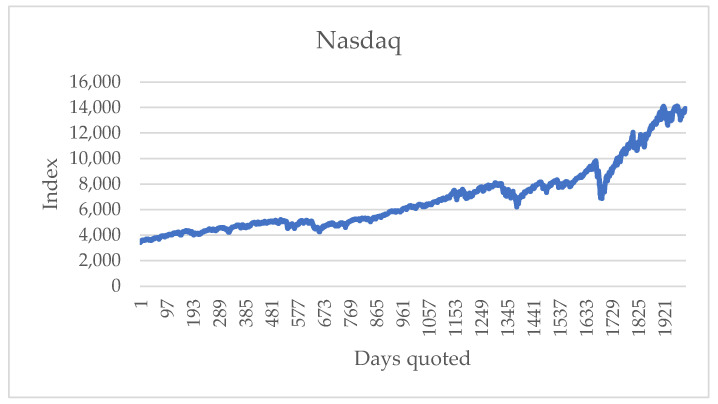
Closing price of the Nasdaq series from 1 July 2013 to 9 June 2021.

**Figure A2:**
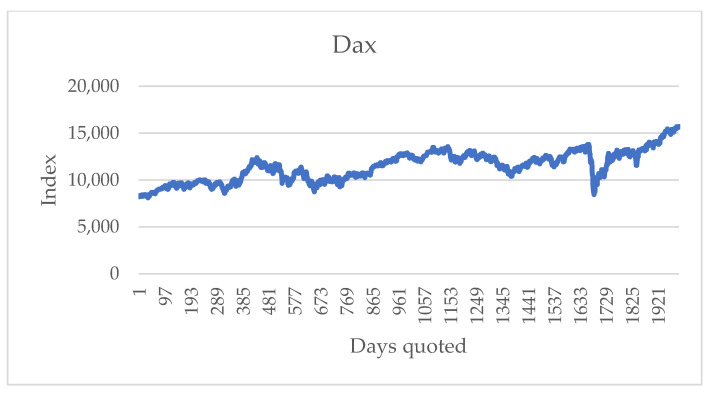
Closing price of the DAX series from 1 July 2013 to 9 June 2021.

**Figure A3:**
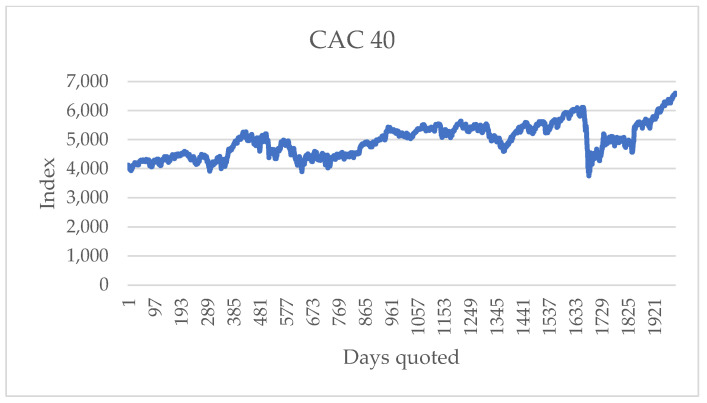
Closing price of the CAC 40 series from 1 July 2013 to 9 June 2021.

**Figure A4:**
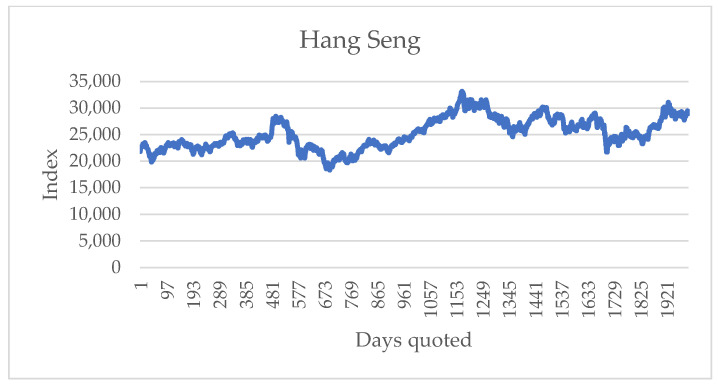
Closing price of the Hang Seng series from 1 July 2013 to 9 June 2021.

**Figure A5:**
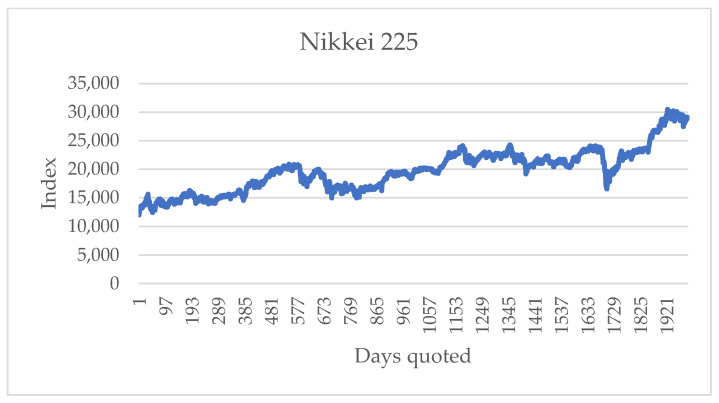
Closing price of the Nikkei series from 1 July 2013 to 9 June 2021.

**Figure A6:**
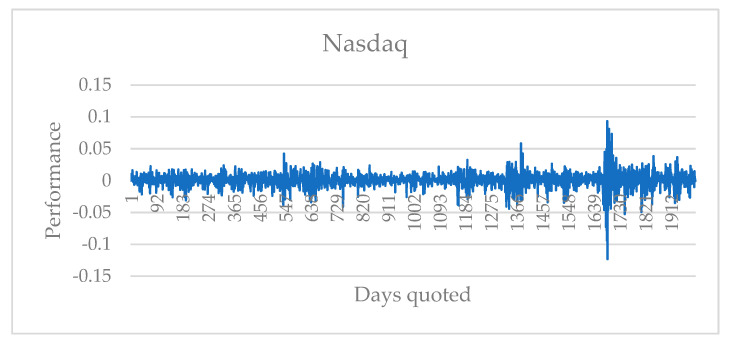
Returns of the Nasdaq series from 1 July 2013 to 9 June 2021.

**Figure A7:**
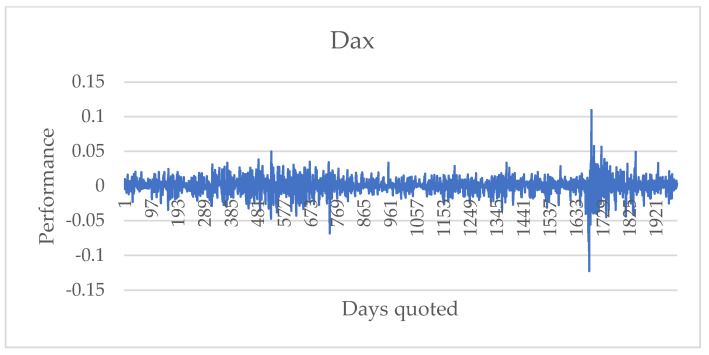
Returns of the DAX series from 1 July 2013 to 9 June 2021.

**Figure A8:**
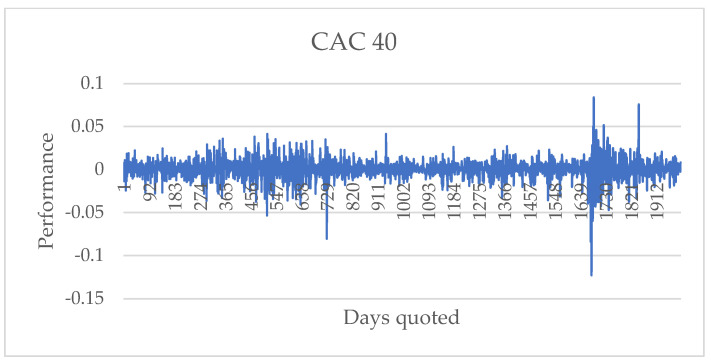
Returns of the CAC 40 series from 1 July 2013 to 9 June 2021.

**Figure A9:**
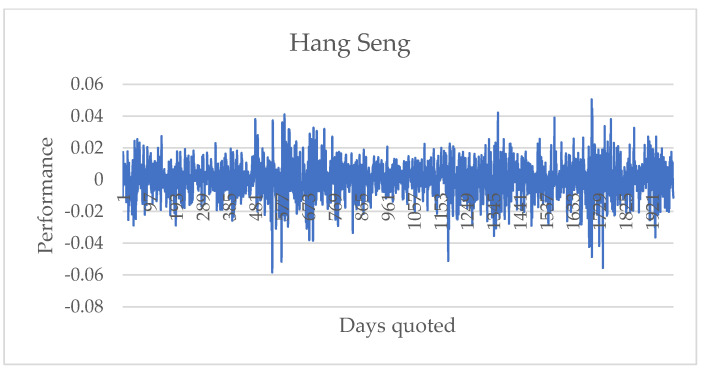
Returns of the Hang Seng series from 1 July 2013 to 9 June 2021.

**Figure A10:**
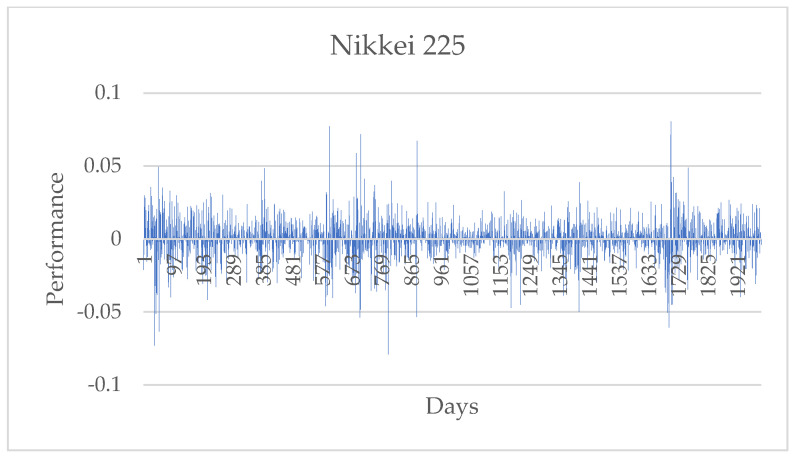
Returns of the Nikkei series from 1 July 2013 to 9 June 2021.

**Figure A11:**
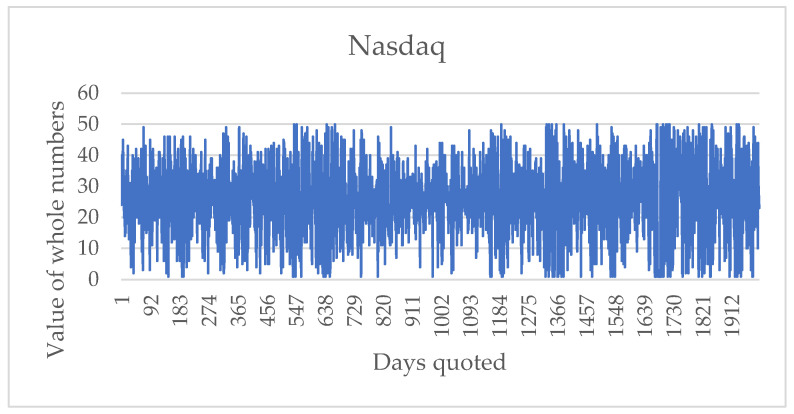
Series of integers of the Nasdaq financial series from 1 July 2013 to 9 June 2021.

**Figure A12:**
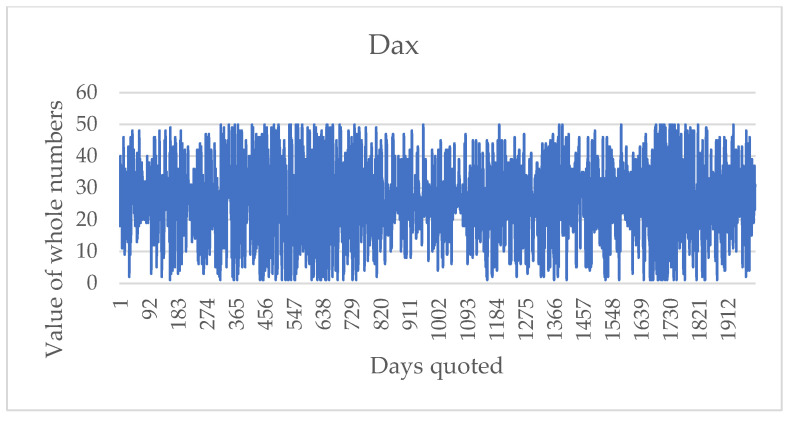
Series of integers of the DAX financial series from 1 July 2013 to 9 June 2021.

**Figure A13:**
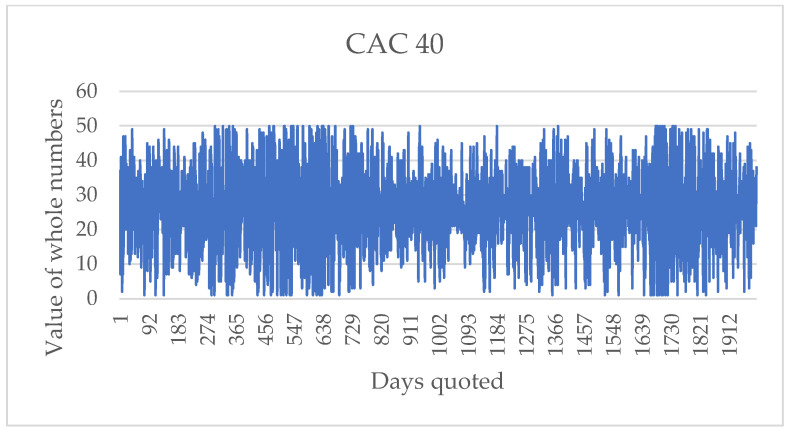
Series of integers of the CAC 40 financial series from 1 July 2013 to 9 June 2021.

**Figure A14:**
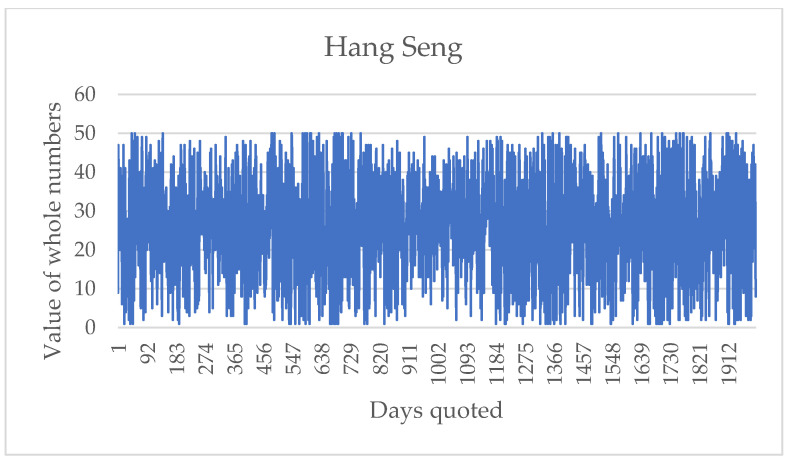
Series of integers of the Hang Seng financial series from 1 July 2013 to 9 June 2021.

**Figure A15:**
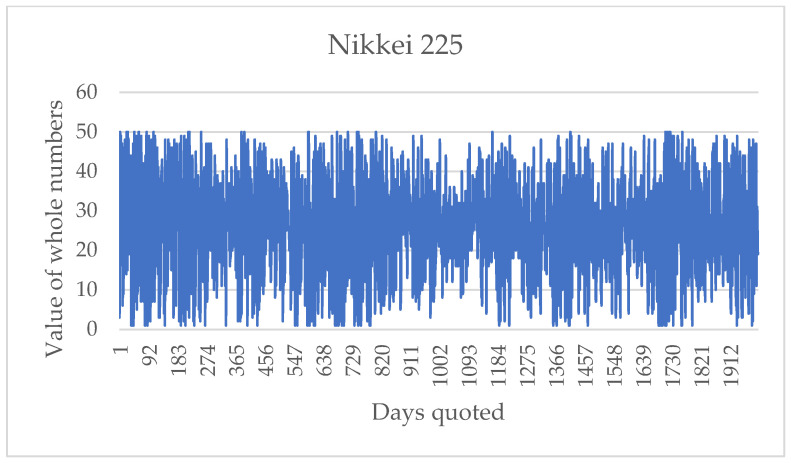
Series of integers of the Nikkei financial series from 1 July 2013 to 9 June 2021.

**Figure A16:**
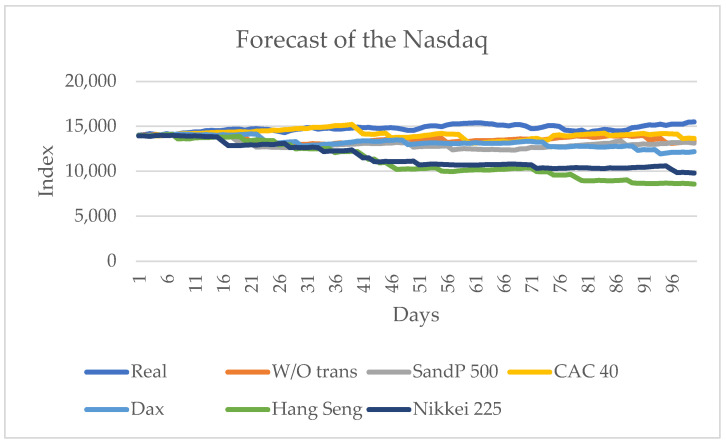
Forecasts for the closing price of the Nasdaq series from 10 June 2021 to 29 October 2021.

**Figure A17:**
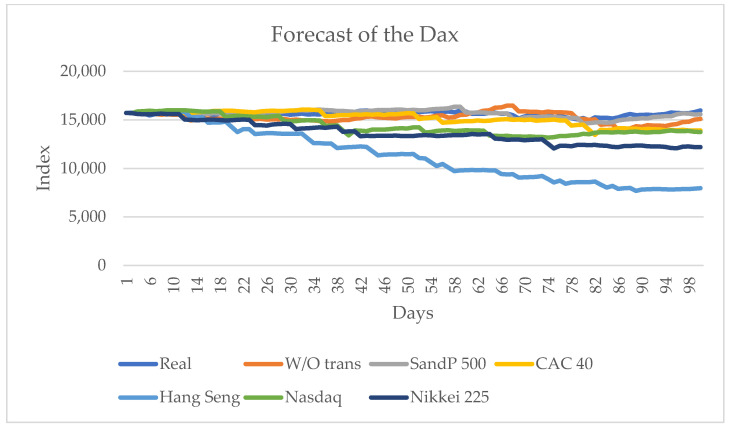
Forecasts for the closing price of the DAX series from 10 June 2021 to 29 October 2021.

**Figure A18:**
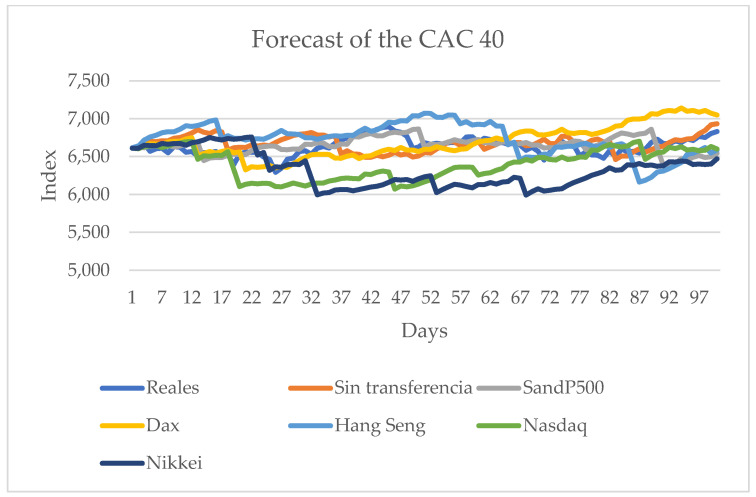
Forecasts for the closing price of the CAC 40 series from 10 June 2021 to 29 October 2021.

**Figure A19:**
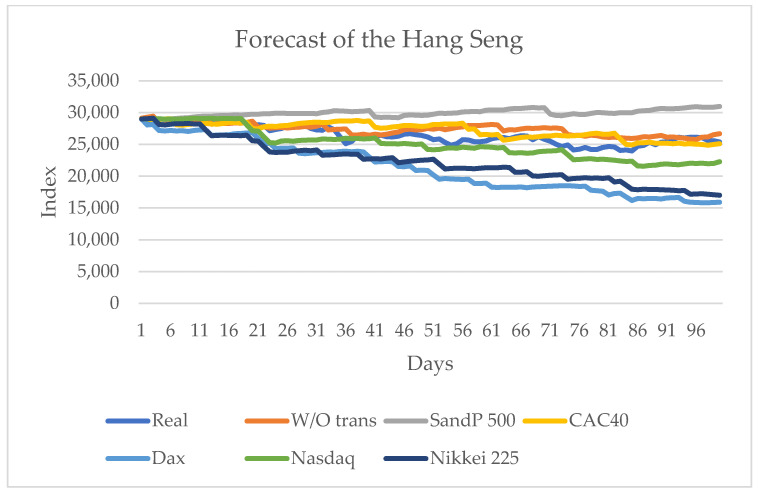
Forecasts for the closing price of the Hang Seng series from 10 June 2021 to 29 October 2021.

**Figure A20:**
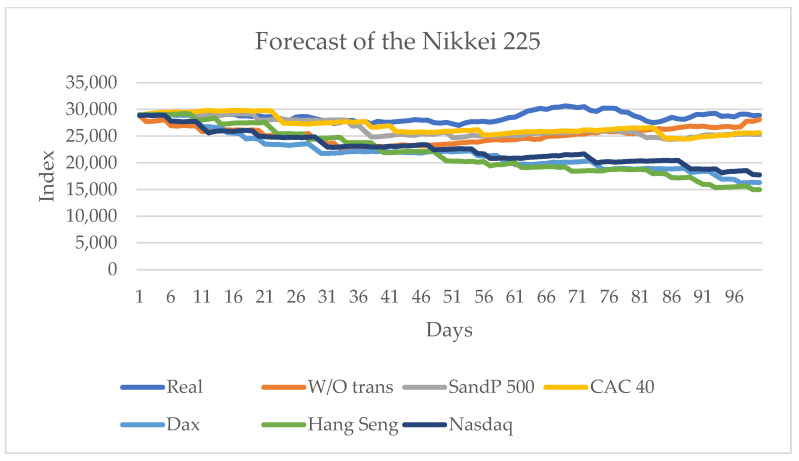
Forecasts for the closing price of the Nikkei series from 10 June 2021 to 29 October 2021.

## Data Availability

https://es-us.finanzas.yahoo.com/mercados/indices-mundo/ (accessed on 30 October 2021).
